# The 24-h Energy Intake of Obese Adolescents Is Spontaneously Reduced after Intensive Exercise: A Randomized Controlled Trial in Calorimetric Chambers

**DOI:** 10.1371/journal.pone.0029840

**Published:** 2012-01-17

**Authors:** David Thivel, Laurie Isacco, Christophe Montaurier, Yves Boirie, Pascale Duché, Béatrice Morio

**Affiliations:** 1 Clermont Université, Université Blaise Pascal, EA3533, UFR STAPS, BP 104, Clermont-Ferrand, France; 2 INRA, UMR1019 Nutrition Humaine, CRNH Auvergne, Clermont-Ferrand, France; 3 Clermont Université, Université d'Auvergne, UMR1019 Nutrition Humaine, Clermont-Ferrand, France; Pennington Biomedical Research Center, United States of America

## Abstract

**Background:**

Physical exercise can modify subsequent energy intake and appetite and may thus be of particular interest in terms of obesity treatment. However, it is still unclear whether an intensive bout of exercise can affect the energy consumption of obese children and adolescents.

**Objective:**

To compare the impact of high vs. moderate intensity exercises on subsequent 24-h energy intake, macronutrient preferences, appetite sensations, energy expenditure and balance in obese adolescent.

**Design:**

This randomized cross-over trial involves 15 obese adolescent boys who were asked to randomly complete three 24-h sessions in a metabolic chamber, each separated by at least 7 days: (1) sedentary (SED); (2) Low-Intensity Exercise (LIE) (40% maximal oxygen uptake, VO_2_max); (3) High-Intensity Exercise (HIE) (75%VO_2_max).

**Results:**

Despite unchanged appetite sensations, 24-h total energy intake following HIE was 6–11% lower compared to LIE and SED (p<0.05), whereas no differences appeared between SED and LIE. Energy intake at lunch was 9.4% and 8.4% lower after HIE compared to SED and LIE, respectively (p<0.05). At dinner time, it was 20.5% and 19.7% lower after HIE compared to SED and LIE, respectively (p<0.01). 24-h energy expenditure was not significantly altered. Thus, the 24-h energy balance was significantly reduced during HIE compared to SED and LIE (p<0.01), whereas those of SED and LIE did not differ.

**Conclusions:**

In obese adolescent boys, HIE has a beneficial impact on 24-h energy balance, mainly due to the spontaneous decrease in energy intake during lunch and dinner following the exercise bout. Prescribing high-intensity exercises to promote weight loss may therefore provide effective results without affecting appetite sensations and, as a result, food frustrations.

**Trial Registration:**

ClinicalTrial.gov NCT01036360

## Introduction

Since obesity in children and adolescents is a public health concern, the search for effective anti-obesity measures has grown in recent years. The effectiveness of multidisciplinary approaches that combine physical activity and dietary strategies has now been recognized. However, the interaction between physical exercise and spontaneous energy consumption has received little consideration, although it could be a key target for controlling daily energy balance. The impact of exercise on subsequent energy intake and appetite sensations has been investigated and reviewed in lean and obese adults [1], [2], [3], [4], [5], [6], but it is still necessary to determine which exercise characteristics such as intensity can lead to a negative energy balance and thus promote weight loss in obese patients, especially within the obese pediatric population.

In normal-weight children, results concerning the impact of acute exercise on subsequent energy intake remain contradictory. Some studies have reported no significant changes in food intake [7], [8], [9], whereas others have shown reduced energy consumption [10], [11]. Very little data is available concerning overweight or obese children and adolescents. Recently, Nemet et al. found increased food consumption in overweight children after resistance, aerobic or swimming sessions of moderate intensity [10]. In contrast, obese adolescents have been shown to significantly reduce their energy intake during lunch and dinner after a single bout of high-intensity cycling (70% VO_2_max), whereas appetite sensations were similar to the resting control session [12]. In addition, the decreased energy consumption was associated with negative daily energy balance. To the best of our knowledge, this last work is the first to simultaneously investigate daily energy intake and daily energy expenditure. However, it used an indirect method based on heart rate and accelerometry recordings for assessing energy expenditure.

Finally, very little data is available at this time concerning the impact of intensive exercise on subsequent macronutrient preferences in obese pediatric populations. Although no modification has been reported in terms of fat and carbohydrate consumption, an increased protein intake has been described in overweight children following moderate-intensity exercise [10]. Understanding the exercise-related regulation of food preferences in obese children and adolescents is of importance for building novel preventive dietary strategies.

In the present study, we hypothesized that the prescribed exercise intensity could be an important factor affecting subsequent energy balance in obese children and adolescents. The study design aimed at determining the differential effects of high vs. moderate intensity exercise compared to a resting condition, on subsequent 24-h energy intake, macronutrient preferences, appetite sensations, energy expenditure and energy balance in obese adolescent boys. Girls were not considered in the present study in order to limit the impact of the variations of female sex hormones during the menstrual cycle on appetite and energy expenditure.

## Methods

The protocol from this trial and supporting CONSORT checklist are available as supporting information; see Checklist S1 and Protocol S1.

### Subjects

Fifteen adolescent boys (Tanner stages 3–4) aged 13.5 (0.9) years were recruited (from January 2010) through the Pediatric Obesity Department of the Children's Medical Center (Centre Médical Infantile) of Romagnat (France). Each participant took part in a screening session to ensure that they met the following criteria: (1) age between 12 and 15 years old; (2) Body Mass Index above the 90^th^ percentile according to international cut-off points [13]; (3) no contraindication to exercising; (4) not taking any medication; and (5) not suffering from claustrophobia. Participants and their legal representatives received information sheets and all provided written informed consent and approval to take part in the study. The study protocol was approved by the relevant Ethical Committee (Comité de Protection des Personnes Sud Est VI, CPPAU814) and registered with the Clinical Trial Protocol Registration System (ClinicalTrials.Gov: NCT01036360).

### Design

Anthropometric characteristics and body composition were measured, and children performed a maximal incremental test to assess maximal oxygen uptake (VO_2_max). Each participant randomly completed three 24-h sessions in indirect calorimetric chambers: a sedentary day (SED); a day with low-intensity exercise (LIE); and a day with high-intensity exercise (HIE). The assignment of the experimental sessions has been counter-balanced for each participants. The adolescents entered the calorimetric chambers at 08:00am where they received a calibrated breakfast. At 11:00am, they were asked to complete a cycling exercise of low- (LIE) or high-intensity (HIE) or to remain inactive (SED). Thirty minutes after the end of the exercise test, an *ad libitum* buffet meal was provided. The adolescents were then asked to remain inactive for the rest of the day, until 07:00pm when a second *ad libitum* buffet meal was provided. Participants then spent a complete night in the calorimetric chambers and went to sleep at 09:30pm. They were awakened at 07:00am on the second day and an *ad libitum* breakfast was distributed. They left the calorimeters at 09:00am. Appetite sensations were recorded throughout the sessions using questionnaires. The experimental sessions were separated by at least seven days and assigned in a randomized order. Indeed, as exposed in overweight or lean adults [14], [15] and obese youths [12], [16], seven days are enough to avoid interferences between experimental sessions.

### Anthropometry and body composition

A digital scale was used to measure body weight to the nearest 0.1 kg, and barefoot standing height was assessed to the nearest 0.1 cm by using a wall-mounted stadiometer. Body Mass Index was calculated as body weight (kg) divided by height squared (m^2^). Waist circumference was measured at a level midway between the last rib and the upper iliac crest. Fat Mass and Fat Free Mass were assessed using dual-energy X-ray absorptiometry (QDR4500A scanner, Hologic, Waltham, MA, USA).

### Maximal oxygen uptake test

VO_2max_ was measured during a graded exhaustive cycling test that was performed at least one week before the experimental sessions. The initial power of 30 W was maintained for three minutes and followed by 15 W increments every 1.5 minutes. Adolescents were strongly encouraged by experimenters throughout the test to perform a maximum effort. Criteria for reaching VO_2max_ were subjective exhaustion with heart rate above 195 beats.min^−1^ and/or Respiratory Exchange Ratio (RER, VCO_2_/VO_2_) above 1.02 and/or a plateau of VO_2_ [17]. An electromagnetically-braked cycle ergometer (Ergoline, Bitz, Germany) was used to perform the test. VO_2_ and VCO_2_ were measured breath-by-breath through a mask connected to O_2_ and CO_2_ analyzers (Oxycon Pro-Delta, Jaeger, Hoechberg, Germany). Calibration of gas analysers was performed with commercial gases of known concentration. Ventilatory parameters were averaged every 30 seconds. ECG was monitored for the duration of the test.

### Calorimetric chambers

Two open-circuit indirect calorimetric chambers were used to continuously measure energy expenditure over 24-h. Each room was equipped with a bed, chair, desk, TV with DVD player, CD player, phone, toilets, washbowl and a cycle ergometer. Gas exchanges were computed from outlet air flow, differences in gas concentration between air entering and leaving the calorimeter, atmospheric pressure, air temperature and hydrometry after correction for the drift and time of response of the gas analyzers and the variations of the volumes of carbon dioxide and oxygen in the calorimeters. Energy expenditure was calculated from VO2 and VCO2 using Weir's equation [18]. Heart rate was continuously measured and recorded by telemetry (Life scope 6; Nikon Kohden, Tokyo, Japan).

### Exercise tests

At 11:00am on LIE and HIE, participants were asked to complete a cycling exercise. The exercise intensity was set at 40% of their VO_2_max during LIE and 75%VO_2_max during HIE. The duration of each exercise was individually calculated so that LIE and HIE tests were isoenergetic for each participant (the targeted energy expenditure was approximately 1400 KJ). The calculation took the number of revolutions per minute and the workload (in watts) into account using individual data obtained during the maximal oxygen uptake test. The targeted heart rate was fixed for each session and monitored by the adolescent using a cardio polar monitor (Polar.Inc-RS800CX Multi). Achievement of the targeted heart rate was also supervised by the investigators and achievement of the targeted energy expenditure was confirmed afterwards using indirect calorimetric measurements.

### Energy intake

#### Fixed breakfast

On the first day of each experimental session (SED, LIE, HIE), the participants received a calibrated breakfast (BF1) whose energy content was calculated in order to reach a null energy balance at 12:00am. This breakfast had to meet the entire morning energy expenditure requirements (rest+exercise) in order to isolate the effect of exercise intensity from that of energy status on subsequent energy intake [19].

#### Ad libitum meal

Lunch time, dinner time and breakfast on day 2 (BF2) were offered *ad libitum* to the participants. The composition of the buffet meal conformed to the adolescents' tastes as determined by the food questionnaire filled in prior to the experimental sessions. Top rated items were avoided to limit overconsumption. The buffets proposed to the participants were identical between adolescents and sessions. Participants were told to eat until satisfied; additional food was available if desired. Food consumption was weighed and recorded by investigators (Bilnut 4.0 SCDA Nutrisoft software, France) to calculate total energy intake. Ad libitum meals methodology and energy intake assessment has been previously detailed [12]. Energy balance was calculated from the difference between energy expenditure and energy intake. The proportion of the total energy intake derived from protein, fat and carbohydrate was calculated thanks to the nutritional software used to assess energy intake.

### Subjective appetite sensations

At regular intervals from 08:00am on day 1 until the next morning, participants were asked to rate their hunger, fullness and desire to eat (prospective-consumption) using visual analogue scales (VAS of 100 mm) whose reliability has been demonstrated [20]. Participants filled in VAS before and after the first breakfast, prior and after the exercise bout, before and after lunch, by mid-afternoon, before and after (immediate and one hour after) dinner time, before and after the second breakfast. Such a method to evaluate appetite feelings has been already used and validated among obese adolescents. [12], [16]

### Statistical analysis

Analyses were performed using Statview 5.0 (SAS Institute, Inc., NC, USA). Results are expressed as mean (standard deviation). The distribution of the data was tested using the Smirnov-Kolmogorov test prior to adapted statistical analysis. Exercise characteristics and the effect of the experimental sessions (SED, LIE, HIE) on energy intake, energy expenditure and energy balance were analyzed using one-way ANOVA with repeated measurements. Macronutrient preferences, subjective appetite, hunger and prospective food consumption were analyzed between conditions using two-way ANOVA with repeated measurements. The Bonferroni test was used for post-hoc analyses. The level of significance was set at 5%.

## Results

### Subject characteristics

Anthropometric characteristics of the population are presented in Table 1. Participants' body mass index averaged 30.7 (4.1) kg/m^2^ and body fat, 38.2 (5.2) % of total body weight. Mean VO_2_max was 3.42 (0.38) L/min.

**Table 1:** Anthropometric characteristics of the adolescents (n = 15).

	Mean (Standard Deviations)
**Age (years old)**	13.5(0.9)
**Weight (Kg)**	84.03(15.4)
**Height (m)**	1.65 (0.10)
**BMI (kg/m^2^)**	30.7 (4.1)
**WC (cm)**	104.3 (9.6)
**Fat Mass (%)**	38.2 (5.2)

Data expressed as mean ± standard deviations; BMI: Body Mass Index; WC: Waist Circumference.

### Energy expenditure

As required by the isoenergetic condition, energy expenditure generated by the two exercise bouts (LIE and HIE) was similar, averaging 1408 (213) and 1387 (198) KJ, respectively (p = 0.87). The mean exercise duration was 30 (3) and 59 (6) min for HIE and LIE, respectively (p<0.001).

Twenty-four-hour energy expenditure (EE) was not significantly different between the three conditions (Table 2). However, EE during the afternoon (12:00am–09:30pm) was significantly lower during HIE (7482(935) KJ) compared with LIE (7984(1038) KJ) and SED (8153(1107) KJ) (p<0.05).

**Table 2:** Energy intake (EI), energy expenditure (EE) and energy balance (EB) in response to sedentary (SED), low-intensity (LIE) or high-intensity (HIE) exercise sessions in obese adolescents (n = 15).

	SED	LIE	HIE
**24 h EI (KJ)**	15145 (2905)	15982 (2442)	14218 (2905)*
**24 h EE (KJ)**	10020 (1300)	10635 (1337)	10363 (1363)
**24 h EB (KJ)**	5125 (1605)	5346 (1105)	3855 (1537)**

Measurements were performed over 24 hours, beginning at 08:00am.

Data expressed as mean ± standard deviations. Significantly different from SED and LIE:

*p<0.05;

**p<0.01.

### Energy intake and energy balance

**Figure 1:**
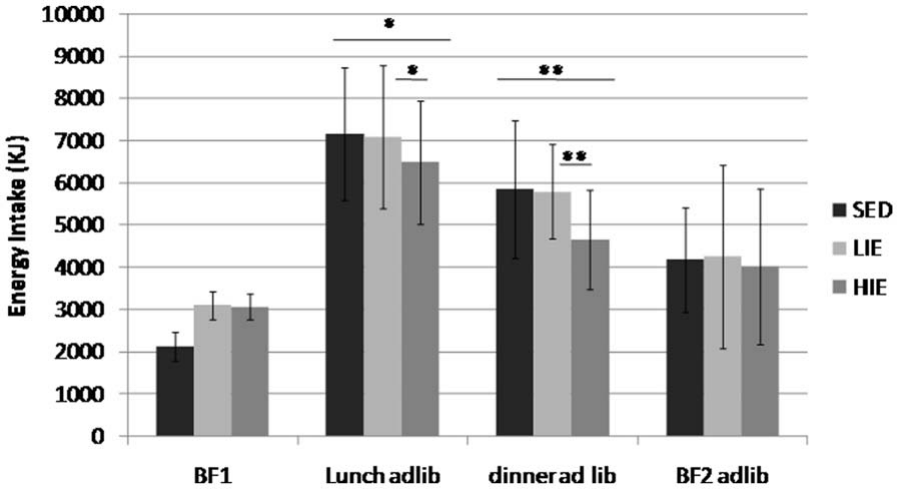
Energy consumption (KJ) distribution between meals for each experimental session (SED: sedentary; LIE: Low-Intensity Exercise; HIE: High-Intensity exercise). Breakfast on day 1 (BF1) was calibrated; lunch, dinner and BF2 (breakfast on day 2) were offered *ad libitum* (adlib). *p<0.05; **p<0.01.

**Figure 2:**
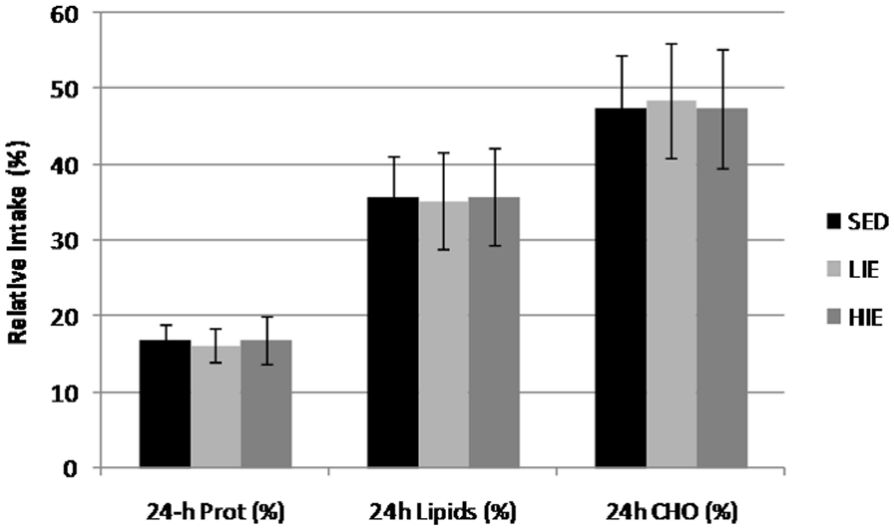
24-h protein (Prot), Lipid and Carbohydrate (CHO) relative intake (%) during each experimental session (SED: Sedentary; LIE: Low-Intensity Exercise; HIE: High-Intensity Exercise).

Energy intake during the *ad libitum* meals (lunch, dinner and breakfast) was significantly altered between conditions (p<0.05). Total energy intake following HIE was 6–11% lower compared to LIE and SED (p<0.05), whereas no differences appeared between SED and LIE (p = 0,21; Table 2). When analyzed separately, energy intake at lunch and dinner was significantly reduced after HIE compared to the two other conditions, whereas energy intake was not different between the three conditions during the *ad libitum* breakfast proposed on day 2 (BF2, Figure 1). Energy intake at lunch was 9.4% and 8.4% lower after HIE, compared to SED and LIE, respectively (p<0.05) (Figure 1). However, energy intake at dinner was 20.5% and 19.7% lower after HIE, compared to SED and LIE, respectively (p<0.01) (Figure 1). As shown in Figure 2, the relative contribution of each macronutrient to 24-h energy intake did not significantly differ between conditions.

Whereas energy balance was not significantly different between conditions and close to null at 12:00am as required by the methodology chosen (SED: −95(24) KJ; LIE: 168(35); HIE: 156(28)), 24-h energy balance was 25–28% lower after HIE in comparison to SED and LIE (p<0.01), and no difference was found between SED and LIE, as presented in Table 2 (p = 0,32).

### Subjective Sensations

**Figure 3:**
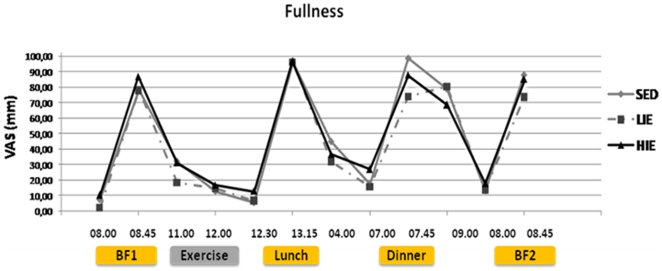
Subjective satiety feeling (Visual Analogue Scale of 100 mm) throughout the experimental sessions (SED: Sedentary; LIE: Low-Intensity Exercise; HIE: High-Intensity Exercise). BF1: calibrated breakfast on day 1; BF2: *ad libitum* breakfast on day 2.

Ratings of subjective hunger, fullness or prospective food consumption were not significantly different between experimental sessions, as illustrated for fullness in Figure 3.

## Discussion

The present study was designed to determine the effect of exercise intensity during an intensive bout of exercise on 24-h energy intake, macronutrient preferences, appetite sensations, energy expenditure and energy balance in obese adolescent boys. The main finding is that a single bout of high-intensity exercise (HIE) induces a decrease in spontaneous daily energy consumption and a reduction in energy balance compared to resting (SED) and low-intensity exercise (LIE) sessions. Importantly, these spontaneous adaptations were not associated with changes in subjective feelings of hunger, satiety or prospective food consumption.

This study is the first to investigate post-exercise regulation of energy intake in obese adolescents placed under balanced energy status, thus isolating the impact of the exercise intensity from the impact of increased energy needs. Adolescents received a calibrated breakfast (BF1) at the beginning of each experimental session so that a null energy balance was reached at midday. As reported by Hagobian et al., post-exercise energy intake depends on the individual's energy status [19]. In fact, when exercise-induced energy expenditure is not replaced by food to equilibrate energy balance, hormonal response is altered in a direction expected to stimulate appetite and restore energy balance [21], [22], [23]. In contrast, when energy is replaced, mixed results are obtained depending on exercise characteristics such as duration and intensity [21], [22], [23], [24], [25]. Because of the completion of isoenergetic exercises during LIE and HIE, the ingested breakfasts during those two sessions were equivalent. However, the absence of exercise during the sedentary condition led to a lower breakfast content during this session. Such a difference in the breakfast energy content between the two exercises sessions and the sedentary one could have influence the adolescents' ad libitum energy intake. However, previous data from our laboratory also underlined reduced energy intake after an acute bout of exercise where the obese adolescents received the same calibrated breakfast on both sedentary and exercise conditions [12], [26].

We effectively recently obtained lower energy consumption in obese adolescent boys and girls after an intensive cycling exercise set at 70% of VO_2_max [12]. The present results showed that only HIE can lead to a spontaneous reduction in energy intake, whereas food consumption following LIE was similar to that after SED (Table 2). The lower energy intake following HIE in the present study could be attributed to a greater mobilization of anorexic peptides such as PYY_3–36_ that has been shown to be associated with exercise intensity in lean [14] and overweight [15] men. Other peptides involved in the regulation of energy intake and appetite, such as glucagon-like peptide 1, cholecystokinin or pancreatic polypeptide, have also been shown to be altered in response to intensive exercise [5]. Finally, increased plasma leptin and/or insulin concentrations could also be involved [27]. It has to be noticed that adolescents showed here high energy consumption during each of the *ad libitum* meals. This overconsumption is certainly mainly due to the environmental conditions of the meals (adolescents were isolated in calorimetric chambers and ate alone) [28], [29]. However, this important food consumption has been observed during the three sessions, the adolescents were then placed under the same experimental conditions.

Our previous work was the first to assess post-exercise energy intake during both lunch and dinner times and to underline a greater reduction in food consumption during dinner in comparison to lunch [12]. A delay in the regulation of energy intake was then suggested with the highest inhibitory effect occurring about seven hours after the exercise bout [12]. The present results corroborate our previous observations with the greater HIE-induced anorexic effect observed once again at the end of the day. To investigate whether this anorexic effect could last longer, the adolescents were offered an *ad libitum* breakfast the next morning. This experiment showed that the HIE-induced spontaneous energy restriction was not sustained overnight.

As protein intake was spontaneously increased in obese children following swimming, aerobic or resistance exercises in comparison to rest [10], we analyzed the respective contribution of macronutrients in the spontaneous food consumption of obese adolescents. Despite a lower consumption of each macronutrient following HIE, no significant difference in the respective contribution of macronutrients to energy intake was found between the three conditions (Figure 2). The lower 24-h energy intake experienced during HIE could not be associated with the alteration of the intake of one specific macronutrient, and exercise intensity does not seem to induce any modification in specific macronutrient preferences in obese adolescents. Corroborating our previous works [12], it is important to stress that these spontaneous adaptations in food consumption were not associated with changes in subjective sensations, suggesting that adolescents decrease their energy balance without food frustrations.

Twenty-four-hour energy expenditure has been precisely assessed by using calorimetric chambers, thus allowing the accurate calculation of energy balance. As shown in Table 2, energy balance was significantly lower during HIE, whereas it was similar between SED and LIE. Although the HIE energy balance is significantly and substantially reduced, it has to be noticed that it remains largely positive. The use of calorimetric chambers that could have limited the adolescents' energy expenditure, combined with the overconsumption favored by the experimental design as exposed previously, are the main reasons explaining such a positive energy balance.

The reduced energy balance during HIE compared with the other sessions was specifically due to the reduction in spontaneous energy intake since energy expenditure during the afternoon following HIE was about 10% lower in comparison to SED and LIE. However, the 24-h energy expenditure was not significantly different between the three conditions. It has been effectively suggested that prescribed exercise may not systemically result in a decrease in energy balance because changes in behavioral aspects, especially reduced spontaneous physical activity, may be compensated for by a decrease in energy expenditure [30], [31], [32]. It has been shown that intensive exercise leads to a higher reduction in daily energy expenditure compared to low-to-moderate exercise in obese adolescents [33]. Thus, if HIE is proposed as a new weight management program to optimize the reduction of the daily energy balance of obese adolescents, it will be necessary to equilibrate the activity program to avoid chronic reduction in spontaneous physical activity, and to limit the adolescents' overconsumption, mainly by controlling the environmental conditions of the meals.

The design of the study and the use of metabolic chambers certainly compose the main strengths of this work, providing highly controlled experimental conditions and an accurate measurement of energy expenditure, the small sample size and the lack of control group being its principal limitations.

In conclusion, despite this reduced sample size, the present study demonstrates the beneficial impact of HIE on 24-h energy balance in obese adolescent boys. The spontaneous decrease in energy intake during lunch and dinner in response to HIE is the main parameter affecting the energy balance. The overconsumption observed in this work, due to the environmental conditions, led to reduced but positive energy balance compared with previously published data [12]. Further studies are required to explore the impact of the meal conditions (alone or with other people, in free living condition or isolated…) on energy consumption in obese adolescents, and then if intensive exercise can decrease energy intake in a same way whatever this meal situation. These results thus open a potential new field of pediatric obesity management, where patients are still able to perform high-intensity exercise with no cardiac restrictions. However, further investigations are now required to examine whether the effects of HIE are maintained under chronic conditions. Although our previous work showed that the impact of HIE on spontaneous food restriction was maintained after a weight loss program [12], questions still remain as to whether the anorexic effect of HIE can be maintained during prolonged training, and whether the reduction in spontaneous physical activity may compensate on the long term for the beneficial impact of HIE on energy balance.

## Supporting Information

Checklist S1 CONSORT Checklist.
(DOC)

Protocol S1 Trial Protocol.
(DOC)
