# Supplementary material for: The 24-h Energy Intake of Obese Adolescents Is Spontaneously Reduced after Intensive Exercise: A Randomized Controlled Trial in Calorimetric Chambers
Source: PLoS One. 2012 Jan 17;7(1):e29840. doi: 10.1371/journal.pone.0029840 (PMC3260158; doi:10.1371/journal.pone.0029840)
Supplement: Protocol S1 — Trial Protocol. (DOC) [file pone.0029840.s002.doc]

| 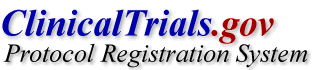 | 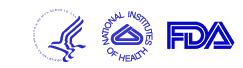 |
| --- | --- |

| **Title**    IND    Sponsor    Summary    Status    Design    Interventions    Conditions    Eligibility    Locations    Citations    Links |
| --- |

| **Title:** | Acute exercise and energy balance regulation; effect of intensity |
| --- | --- |

| [Unique Protocol ID:](http://prsinfo.clinicaltrials.gov/definitions.html#PrimaryId) | AU 814 |
| --- | --- |
| [Brief Title:](http://prsinfo.clinicaltrials.gov/definitions.html#BriefTitle) | Acute exercise and energy balance regulation; effect of intensity |
| [Official Title:](http://prsinfo.clinicaltrials.gov/definitions.html#OfficialTitle) | Regulation of the energy balance following an acute exercise in metabolic chambers: effect of the exercise intensity, age and weight status |
| [Secondary ID's:](http://prsinfo.clinicaltrials.gov/definitions.html#SecondaryIds) (***One ID per line***)  Definition: Other identification numbers assigned to the protocol, including any applicable NIH grant numbers. Provide up to 5 Secondary ID Numbers. Example: NCI-793-0115D | AOI 2009 BOUTELOUP |

| Title    IND    **Sponsor**    Summary    Status    Design    Interventions    Conditions    Eligibility    Locations    Citations    Links |
| --- |

| [Sponsor:](http://prsinfo.clinicaltrials.gov/definitions.html#LeadSponsor)  **Definition**: Name of sponsoring organization that takes responsibility for and initiates a clinical investigation | **CHU Clermont Ferrand** |
| --- | --- |
| [Collaborators:](http://prsinfo.clinicaltrials.gov/definitions.html#Collaborators) (***Enter up to 10 agencies, one agency per line***)  **Définition** : Full names of all organizations co-sponsoring and/or providing financial support for the protocol. The data provider is responsible for confirming all collaborators before listing them. Provide up to 10 full names of collaborating organizations | Regional Program for Clinical Research 2009 (PHRC 2009)  Thermal Institution of Brides les Bains (FRANCE) |

REVIEW BOARD

| [Board Approval Number:](https://register.clinicaltrials.gov/prs/html/definitions.html#HumanSubject) ***** | AU814 |
| --- | --- |
| [Board Name:](https://register.clinicaltrials.gov/prs/html/definitions.html#IRBName) ***** | CPP Sud Est VI |
| [Board Chair:](https://register.clinicaltrials.gov/prs/html/definitions.html#IRBChair) ***** (Not made public) | | Name: | Pr Bazin | | --- | --- | | Business Phone: | Extension: | | Business Email: |  | | Business Address: |  | |

| Title    IND    Sponsor    **Summary**    Status    Design    Interventions    Conditions    Eligibility    Locations    Citations    Links |
| --- |

| [Brief Summary:](http://prsinfo.clinicaltrials.gov/definitions.html#BriefSummary)  **Definition**: Short description of the primary purpose of the protocol intended for the lay public. Include a brief statement of the study hypothesis. | Physical activity is mainly considered and use for its impact on energy expenditure in the treatment of obesity, and less is known concerning its indirect effects on energy intake. The aim of this work is to clarify the impact of an acute bout of exercise, depending on its intensity (high vs low), on the following energy intake and nutrient utilization, in obese and non-obese boys and men. |
| --- | --- |
| [Detailed Description:](http://prsinfo.clinicaltrials.gov/definitions.html#DetailedDescription)  **Definition:** Extended description of the protocol, including information not already contained in other fields, such as comparison(s) studied. | 20 adolescent boys (10 obese and 10 leans) and 20 men (10 obese and 10 lean) will complete a sub-maximal test on a ergo cycle to draw their linear relationship between VO_2_ and Fc. Then they will enter a metabolic chamber three times, for 24 hours each. A first session will be considered as sedentary, and volunteers will remain inactive. During the second and third session, they will have to complete a cycling test generating an energy expenditure of 400 Kcal, once at low intensity (40%VO_2_max) and once at high intensity (70% VO_2_max). Those three sessions will be realized in a randomized order with an interval of at least 7 days and urinary collection over the 24hours will be done during all the sessions. At the beginning of the protocol, blood samples will be taken, body composition assessed by DXA and adiposity location evaluated by MNR. |

| Title    IND    Sponsor    Summary    **Status**    Design    Interventions    Conditions    Eligibility    Locations    Citations    Links |
| --- |

| [Study Phase:](http://prsinfo.clinicaltrials.gov/definitions.html#StudyPhase) | **N/A** | |
| --- | --- | --- |
| [Study Type:](http://prsinfo.clinicaltrials.gov/definitions.html#StudyType) | [Observational](http://prsinfo.clinicaltrials.gov/definitions.html#Observational) | |
| [Overall Study Status:](http://prsinfo.clinicaltrials.gov/definitions.html#OverallStatus)  **Definition:** Overall protocol accrual activity for the protocol | **Recruiting**: participants are currently being recruited and enrolled | |
| [Record Verification Date:](http://prsinfo.clinicaltrials.gov/definitions.html#VerificationDate)  Definition: Date the protocol information, including recruiting status, was last verified, whether changes were made or not. | **(12-2009)** | |
| **Key Trial Dates** | | |
| [Study Start Date:](http://prsinfo.clinicaltrials.gov/definitions.html#StartDate)  Definition: Date that enrollment to the protocol begins | | **(12-2009)** |
| [Last Follow-Up Date:](http://prsinfo.clinicaltrials.gov/definitions.html#LastFollowUpDate)  Definition: Date that follow-up with all study participants is complete | | **(12-2009)** |
| [Data Entry Closure Date:](http://prsinfo.clinicaltrials.gov/definitions.html#LastDataEntryDate)  Definition: Date that data submission for the study is complete. | | **(06-2011)** |
| [Study Completion Date:](http://prsinfo.clinicaltrials.gov/definitions.html#CompletionDate)  Definition: Expected or actual date that analysis is concluded for the protocol. | | **(06-2011)** |

**Quand** **Study Type** [Observational](http://prsinfo.clinicaltrials.gov/definitions.html#Interventional)

| Title    IND    Sponsor    Summary    Status    **Design**    Interventions    Conditions    Eligibility    Locations    Citations    Links |
| --- |

| [Purpose:](http://prsinfo.clinicaltrials.gov/definitions.html#IntPurpose)  Reason for the protocol   - - - **Treatment**: protocol designed to evaluate one or more interventions for treating a disease, syndrome, or condition     - **Prevention**: protocol designed to assess one or more interventions aimed at preventing the development of a specific disease or health condition     - **Diagnosis**: protocol designed to evaluate one or more interventions aimed at identifying a disease or health condition     - Educational/Counseling/Training: protocol designed to assess one or more interventions in an educational, counseling, or training environment | *Prevention* |
| --- | --- |
| [Allocation:](http://prsinfo.clinicaltrials.gov/definitions.html#IntAllocation)  Participant selection   - - - **Randomized Controlled Trial**: participants are assigned to intervention groups by chance     - **Nonrandomized Trial**: participants are expressly assigned to intervention groups | *Randomized control trial* |
| [Masking:](http://prsinfo.clinicaltrials.gov/definitions.html#IntMasking)  knowledge of intervention assignments   - - - **Open**: no masking is used. All involved know the identity of the intervention assignment.     - **Single Blind**: participants are unaware of the intervention assignment; investigators are aware.     - **Double Blind**: both participants and investigators are unaware of the intervention assignment | *Open* |
| [Control:](http://prsinfo.clinicaltrials.gov/definitions.html#IntControl)  Nature of the intervention control   - - - **Placebo**: participants may receive only placebo throughout the course of the protocol     - **Active**: participants may receive some form of treatment (e.g., standard treatment) in place of the intervention under investigation     - **Uncontrolled**: no controls are used     - **Historica**l: the control consists of results from past studies     - **Dose Comparison**: participants may receive one of several doses of the intervention | *Active* |

| [Assignment:](http://prsinfo.clinicaltrials.gov/definitions.html#IntAssignment)  - intervention assignments   - - - **Single Group**: all participants receive the same intervention throughout the protocol     - **Parallel**: participants receive an intervention throughout the protocol     - **Cross-over**: participants may receive different interventions sequentially during the protocol     - **Factorial**: participants may receive no intervention, some intervention, or multiple interventions simultaneously     - **Expanded Access**: includes treatment IND protocols | *Single group* |
| --- | --- |
| [Endpoints:](http://prsinfo.clinicaltrials.gov/definitions.html#IntEndpoints)  overall outcome that the protocol is designed to evaluate. Select one.   - - - **Safety**: show if the drug is safe under conditions of proposed use     - **Efficacy**: measure of an intervention's influence on a disease or health condition     - **Safety/Efficacy**     - **Bio-equivalence**: scientific basis for comparing generic and brand name drugs     - **Bio-availability**: rate and extent to which a drug is absorbed or otherwise available to the treatment site in the body     - **Pharmacokinetics**: the action of a drug in the body over a period of time including the process of absorption, distribution and localization in tissue, biotransformation, and excretion of the compound     - **Pharmacodynamics**: action of drugs in living systems     - **Pharmacokinetics/dynamics** | *Efficacy* |
| [Primary Outcomes:](http://prsinfo.clinicaltrials.gov/definitions.html#Outcomes)  **Definition:** The specific measure that will be used to determine the effect of the intervention(s). The description should include the time at which the measure will be taken.  **Examples:** all cause mortality at one year; score on a depression rating scale at 6 weeks | The amount of energy intake over the 24 hours is the primary outcome.  It will be assessed during the 3 experimental conditions:  -Sedentary  - Low intensive exercise  - High intensive exercise |
| [Secondary Outcomes:](http://prsinfo.clinicaltrials.gov/definitions.html#Outcomes)  **Definition:** Other measures that will be used to evaluate the intervention(s), and that are specified in the protocol. The description should include the time at which the measures will be taken.  **Examples:** cardiovascular mortality at 6 months; functional status at 4 weeks | Appetite feeling and substrate utilization are the two second outcomes.  It will be assessed during the 3 experimental conditions:  -Sedentary  - Low intensive exercise  - High intensive exercise |

**Quand** **Study Type** [Observational](http://prsinfo.clinicaltrials.gov/definitions.html#Observational)

| Title    IND    Sponsor    Summary    Status    **Design**    Interventions    Conditions    Eligibility    Locations    Citations    Links |
| --- |

| [Purpose:](http://prsinfo.clinicaltrials.gov/definitions.html#IntPurpose)  Reason for the protocol   - **Natural History**: protocol designed to investigate a disease or condition through observation under natural conditions (i.e., without intervention) - **Screening**: protocol designed to assess or examine persons or groups in a systematic way to identify specific markers or characteristics (e.g., for eligibility for further evaluation) - **Psychosocial**: protocol designed to observe the psychosocial impact of natural events | *Natural history* |
| --- | --- |
| **Duration** *****  length of protocol   - **Longitudinal**: studies in which participants are evaluated over long periods of time, typically months or years - **Cross-sectional**: studies in which participants are evaluated over short periods of time, typically up to 10 weeks | *Cross sectional* |
| **Selection** *****  sample selection   - **Convenience Sample**: participants or populations are selected due to ease of recruitment - **Defined Population**: participants or populations are selected based on predefined criteria - **Random Sample**: participants or populations are selected by chance - **Case Control**: participants or   populations are selected to match the  control participants or populations in all  relevant factors except for the disease;  only the case participants or populations  have the disease | *Defined population* |
| **Timing** ***** - time of protocol   - **Retrospective**: a protocol that observes   events in the past   - **Prospective**: a protocol that observes events in real time (may occur in the future) - **Both**: a protocol that combines   retrospective and prospective observation | *prospective* |

| Title    IND    Sponsor    Summary    Status    Design    **Interventions**    Conditions    Eligibility    Locations    Citations    Links |
| --- |

Provide a type and specific name for each intervention.   For drugs, please use the generic name if known.

| [Intervention Type:](http://prsinfo.clinicaltrials.gov/definitions.html#InterventionType) | *Select one* **per intervention** |
| --- | --- |

| [Intervention Name:](http://prsinfo.clinicaltrials.gov/definitions.html#InterventionName)  generic name of the precise intervention being studied  Examples:  Zidovudine (drug) Self-hypnotic relaxation (behavior |  |
| --- | --- |

| Title    IND    Sponsor    Summary    Status    Design    Interventions    **Conditions**    Eligibility    Locations    Citations    Links |
| --- |

Specify the primary condition or disease being studied.

| [Conditions:](http://prsinfo.clinicaltrials.gov/definitions.html#Conditions) (Enter 1 to 5 conditions, one per line)  **Definition:** Primary diseases or conditions being studied, using the National Library of Medicine's Medical Subject Headings (MeSH) controlled vocabulary. The conditions are used to index studies in ClinicalTrials.gov. Select up to five disease or condition terms. | Obesity |
| --- | --- |
| [Keywords:](http://prsinfo.clinicaltrials.gov/definitions.html#Keywords) (One per line)  Definition: Words or phrases that best describe the protocol. Keywords help users find studies in the database. Use NLM's Medical Subject Heading (MeSH) controlled vocabulary terms where appropriate. Be as specific and precise as possible. Avoid acronyms and abbreviations. | Obesity  Acute exercise  Intensity of exercise  Energy balance regulation  Appetite |

| Title    IND    Sponsor    Summary    Status    Design    Interventions    Conditions    **Eligibility**    Locations    Citations    Links |
| --- |

| [Eligibility Criteria:](http://prsinfo.clinicaltrials.gov/definitions.html#EligibilityCriteria) | Inclusion criteria :  - Male  - Adults between 18 to 30 years old and adolescents between 12 and 15yo  - Body mass index :  - lean adults : 20 <BMI< 25 kg.m²  - obese adults : 30<BMI<38 kg.m²  - lean adolescents: BMI <90^th^ percentile  - obese adolescents: BMI>97^th^ percentile  - Affiliated to National Health Insurance  - Subject giving his written informed consent  - Subject considered as normal after clinical examination and medical questionnaire.  Exclusion criteria :  - Chronic pathologies : cardiovascular diseases, cancer, chronic inflammation diseases, renal, intestinal impairments  - Refusal to be registered on the National Volunteers Data file  - Being in exclusion on the National Volunteers Data file  - Practising intensive physical exercise  - Heavy consumer of alcohol or/and tobacco  - Previous medical and/or surgery judged by the investigator as incompatible with this study |
| --- | --- |
| [Gender:](http://prsinfo.clinicaltrials.gov/definitions.html#EligibilityGender)  Definition: Physical gender of individuals who may participate in the protocol. Select one.   - - **Both**: both female and male participants are being studied   - **Female**: only female participants are being studied   - **Male**: only male participants are being studied | male |
| [Age Limits:](http://prsinfo.clinicaltrials.gov/definitions.html#EligibilityMinAge) | | Minimum:   adults : 18yo  Adolescents : 12yo |  | Maximum: adults : 30 yo  Adolescents : 15 yo |  | | --- | --- | --- | --- | |
| [Participants:](http://prsinfo.clinicaltrials.gov/definitions.html#HealthyVolunteers) | **Accepts Healthy Volunteers?** YES |
| [Expected Total Enrollment:](http://prsinfo.clinicaltrials.gov/definitions.html#TotalEnrollment)  **Definition:** Estimated number of participants to be studied | 40 |

| Title    IND    Sponsor    Summary    Status    Design    Interventions    Conditions    Eligibility    **Locations**    Citations    Links |
| --- |

| [Facility:](http://prsinfo.clinicaltrials.gov/definitions.html#Facility) Name: Full name of the organization where the protocol is being conducted. Examples: UCLA Eye Institute; Springfield Memorial Hospital   - - City   - State/Province   - Postal Code   - Country | Name: Centre de Recherche en Nutrition Humaine Auvergne  City: Clermont Ferrand State/Province: Postal Code: 63000 Country: France | |
| --- | --- | --- |
|  | | |
| [Recruitment Status:](http://prsinfo.clinicaltrials.gov/definitions.html#FacilityStatus) | | recruiting |
|  | | |
| [Facility Contact:](http://prsinfo.clinicaltrials.gov/definitions.html#FacilityContact)   - - First Name   - Middle Initial   - Last Name   - Degree   - Phone: office phone of the facility contact person. Use the format 123-456-7890 within the United States and Canada. Otherwise, provide the country code.   - Ext: phone extension, if needed   - Email: electronic mail address of the facility contact person | | Principal investigator :  Dr Bouteloup Corinne  [cbouteloup@chu-clermontferrand.fr](mailto:ksoulier@chu-clermontferrand.fr)  Study Director  Pr Duché Pascale  +33 (0)4 73 40 54 88  Pascale.DUCHE@univ-bpclermont.fr |

| Title    IND    Sponsor    Summary    Status    Design    Interventions    Conditions    Eligibility    Locations    **Citations**    Links |
| --- |

  Provide the unique PubMed Identifier for the citation.

[Search for a citation](http://www.ncbi.nlm.nih.gov/entrez/query/static/citmatch.html) in MEDLINE, using the PubMed browser.

| [MEDLINE Identifier:](http://prsinfo.clinicaltrials.gov/definitions.html#PubMedId) |  |
| --- | --- |
| [Results Reference?](http://prsinfo.clinicaltrials.gov/definitions.html#IsResultsRef) |  |

| Title    IND    Sponsor    Summary    Status    Design    Interventions    Conditions    Eligibility    Locations    Citations    **Links** |
| --- |

| [URL:](http://prsinfo.clinicaltrials.gov/definitions.html#URL) |  |
| --- | --- |
| [Description:](http://prsinfo.clinicaltrials.gov/definitions.html#LinkDescription) |  |
